# Causes and predictors of failed same-day home discharge following primary hip and knee total joint arthroplasty: a Canadian perspective

**DOI:** 10.1177/11207000221111101

**Published:** 2022-07-17

**Authors:** Aaron M Gazendam, Daniel Tushinski, Mansi Patel, Kamal Bali, Danielle Petruccelli, Mitchell Winemaker, Justin DeBeer, Leslie Gillies, Katie Best, Jennifer Fife, Thomas J Wood

**Affiliations:** 1 McMaster University, Hamilton, ON, Canada; 2 Department of Surgery, McMaster University, Hamilton, ON, Canada; 3 Orthopaedic Surgery, Hamilton Health Sciences, Hamilton, ON, Canada

**Keywords:** Failed discharge, same-day home, total joint arthroplasty

## Abstract

**Purpose:**

Same-day home (SDH) discharge in total joint arthroplasty (TJA) has increased in popularity in recent years. The objective of this study was to evaluate the causes and predictors of failed discharges in planned SDH patients.

**Methods:**

A consecutive cohort of patients who underwent total knee (TKA) or total hip arthroplasty (THA) that were scheduled for SDH discharge between 01 April 2019 and 31 March 2021 were retrospectively reviewed. Patient demographics, causes of failed discharge, perioperative variables, 30-day readmissions and 6-month reoperation rates were collected. Multivariate regression analysis was undertaken to identify independent predictors of failed discharge.

**Results:**

The cohort consisted of 527 consecutive patients. 101 (19%) patients failed SDH discharge. The leading causes were postoperative hypotension (20%) and patients who were ineligible for the SDH pathway (19%). 2 individual surgeons, later operative start time (OR 1.3; 95% CI, 1.15–1.55; *p* = 0.001), ASA class IV (OR 3.4; 95% CI, 1.4–8.2; *p* = 0.006) and undergoing a THA (OR 2.0; 95% CI, 1.2–3.1, *p* = 0.004) were independent predictors of failed SDH discharge. No differences in age, BMI, gender, surgical approach or type of anaesthetic were found (*p* *>* 0.05). The 30-day readmission or 6-month reoperation were similar between groups (*p* *>* 0.05).

**Conclusions:**

Hypotension and inappropriate patient selection were the leading causes of failed SDH discharge. Significant variability existed between individual surgeons failed discharge rates. Patients undergoing a THA, classified as ASA IV or had a later operative start time were all more likely to fail SDH discharge.

## Introduction

Total joint arthroplasty (TJA) is an effective intervention for the management of end-stage osteoarthritis that is refractory to non-surgical treatments. Notably, in Canada, 62,016 hip replacements and 75,345 knee replacements were performed in 2018–2019.^1^ In relation to 5 years earlier, there has been a surge in the demand for TJA, with volume increases of 20.1% and 22.5% for total hip arthroplasty (THA) and total knee arthroplasty (TKA), respectively.^1^ This increased demand is expected to encumber healthcare services and resources, particularly if patients require prolonged postoperative monitoring or an inpatient hospital stay.^2^

Changes in postoperative management, due to advances in perioperative care including short-acting neuraxial anaesthetics, long-acting local anaesthetics, and blood loss management, have decreased the need for overnight hospitalisation postoperatively in TJA.^3^ As a result, there has been an increase in the number of TJA procedures performed in an outpatient setting, also referred to as same-day home discharge (SDH).^2,4,5^ Same-day home arthroplasty alleviates financial constraints and has been demonstrated to be a safe alternative to traditional inpatient TJA procedures.^4^ Financial analyses have demonstrated that if outpatient THA is performed for just 30% of the 250,000 procedures performed annually, it would save $300 million in billing charges and $87 million in reimbursements in the USA.^6,7^

Proper patient selection, education and a well-defined clinical pathway are keys for successful SDH in patients undergoing TJA.^3,8^ Specifically, the success of SDH is contingent on a multidisciplinary team-based approach comprised of standardised and comprehensive preoperative and postoperative protocols, rigorous patient selection, and preoperative patient education.^8^ A number of factors including increased age, elevated body mass index (BMI) and other medical comorbidities, place certain TJA patients in a higher risk category and make them more likely to fail SDH discharge.^3,8^ Despite knowledge of this, failures of SDH continue to occur indicating that current risk stratification and patient selection algorithms remain incomplete.^9^ Additionally, the majority of literature evaluating predictive factors associated with SDH TJA comes from insurance-based healthcare systems.^10,11^ An evaluation of SDH TJA in a public, single-payer Canadian healthcare system is required given the differences in patient population and institutional constraints.^12^

Establishing a deeper understanding of the causes of failed SDH and potential predictive factors in this population is critical to inform both clinicians and patients. Accordingly, this study aims to: (1) identify primary reasons for failed SDH discharge; and (2) evaluate patient and perioperative factors associated with failed SDH in patients undergoing elective primary TJA in a hospital setting.

## Methods

### Study design

A retrospective review was undertaken to identify the rates, causes and predictors of failed SDH in patients undergoing elective primary TJA. This study followed the Strengthening the Reporting of Observational Studies in Epidemiology (STROBE) guidelines for reporting of observational studies.^13^ Research ethics approval was obtained through the Hamilton Integrated Research Ethics Board.

### Setting

All patients who underwent primary TJA and were scheduled to undergo SDH at a high-volume academic orthopaedic centre between 01 April 2019 and 31 March 2021, were included for review.

### Participants

All patients who underwent primary TJA and were scheduled for SDH at our institution were included in the analysis. Given that the majority of SDH pathways, including at our institution, include both THA and TKA patients within 1 standardised pathway, we felt it was appropriate to include both groups in the analysis.^3,11,14^ Patients were excluded if they were undergoing revision surgery or undergoing surgery for fracture or malignancy. Patients were excluded if inadequate data was available for analysis. The institutional eligibility for SDH TJA is outlined in Table 1.

**Table 1.** Eligibility criteria for same-day home pathway.

Inclusion	Exclusion
● Unilateral primary THA or TKA	● Cardiopulmonary disease necessitating inpatient postoperative monitoring
● BMI <40 kg/m^2^	● Contraindication to tranexamic acid
● “Care partner” available to stay with patient for minimum of 48 hours post-op	● Bleeding disorders
	● Chronic opioid or benzodiazepine use
● No history of OSA	● Anticoagulant use (excluding ASA)
● Preoperative Hb ⩾120 g/L for females or ⩾130 g/L for males	● Poor preoperative mobility level
● Live within 30 minutes of hospital with emergency services	● Cognitive issues precluding the ability to understand instructions
● Able to communicate in English	● Patient unable to attend preoperative visits

THA, total hip arthroplasty; TKA, total knee arthroplasty; BMI, body mass index; OSA, obstructive sleep apnoea; Hb, haemoglobin; ASA, acetylsalicylic acid.

### Criteria for discharge

The criteria for discharge was based on the modified Aldrete score.^15^ A minimum Aldrete score of 15 as determined by the same-day surgery nursing staff was required for discharge. An assessment by the physiotherapist was required prior to discharge. Patients were required to: (1) transfer in/out of bed safely; (2) mobilise safely; (3) go up and down stairs safely (if stairs in the home); and (4) void prior to discharge if spinal anaesthetic utilised.

### Data sources and variables

Patient demographic data and surgical data were obtained from the prospectively collected database for arthroplasty patients at our institution. Patient demographic included age, gender, BMI, ASA and history of previous TJA of another joint. Surgical data included operative procedure performed (TKA or THA), time of surgery and anaesthetic used. Patients were also categorised based on treating surgeon. Failed SDH was defined as an inpatient admission requiring overnight hospital stay. The reason(s) for failed discharge and ultimate inpatient hospital length of stay was recorded. Causes for failed discharge were broadly categorised into 5 groups:

(1) Pain crisis postoperatively
(2) Falls risk: spinal not fully regressed, requiring supervision for mobility or transfers, decreased weight-bearing status, fatigue with ambulation
(3) Requiring O2 monitoring: undiagnosed obstructive sleep apnoea (OSA), desaturating intraoperatively, history of other respiratory conditions requiring monitoring at the recommendation of anaesthesia
(4) Not medically stable: hyper/hypotension, chest pain, urinary retention, requiring supplemental O2, anaemia, recalcitrant postoperative nausea and vomiting
(5) Not eligible for SDH: history of OSA, late OR start, no support person at home.

Unplanned 30-day readmissions and 6-month reoperations or manipulations under anaesthesia were obtained through the Canadian Institute for Health Information. This ensured that readmissions occurring not at our institution were captured. The cause and nature of the readmission and/or reoperation were recorded.

Demographic data were reported using descriptive statistics, with mean and standard deviation (SD) or median and interquartile range (IQR), as appropriate, depending on data distribution. Continuous outcomes between groups were compared using a Student’s *t*-test and dichotomous outcomes between groups were compared using a Pearson’s chi-square test. All variables that yielded a statistically significant *p*-value (*p* *<* 0.05) from the bivariate analysis were included in the multivariable logistic regression that was performed to assess whether risk factors were independently associated with a failed SDH discharge. Outcomes of the multivariate logistic regression were presented with odds ratios (OR) and 95% confidence intervals (CI). All analyses were performed using SPSS (IBM SPSS Statistics for Mac, V26). A value of *p* *<* 0.05 was considered to be significant for all analyses.

## Results

### Cohort characteristics

A consecutive series of 527 patients undergoing primary TJA, scheduled for SDH discharge, were identified and included (Table 2). The study cohort was comprised of 306 females (58%) and 221 males (42%) with a mean age at the time of surgery of 64 (±8) years and a preoperative BMI of 29.5 (±5) kg/m^2^. The median ASA score was III (range I–IV). There were 272 TKAs and 255 THAs performed, with 50 patients having undergone a previous TKA or THA. The average surgical start time for patients included in the cohort was 9:34 a.m. (±88 minutes). There was a total of 10 readmissions within 30 days of the index surgery and 11 reoperations within a 6-month window of the index surgery (Appendix 1). Regarding THA, 124 patients underwent the lateral approach, 114 via the didrect anterior approach (DAA) and 17 *via* the posterior approach (Appendix 2).

**Table 2.** Cohort characteristics.

Variable	Entire cohort (*n* = 527)	Successful discharge (*n* = 426)	Failed discharge (*n* = 101)	*p*-Value
Age (years)	64 ± 8	64 ± 7.9	64 ± 8.3	0.983
BMI (kg/m^2^)	29.5 ± 5	29.4 ± 4.9	29.8 ± 5	0.445
Gender (M/F)	221/306	177/249	44/57	0.712
ASA Class				**0.031**
I	7	7	0	
II	114	93	21	
III	381	311	70	
IV	25	15	10	
Previous TJA	50	47	3	**0.013**
Operation				**0.007**
TKA	272	232	40	
THA	255	194	61	
Anaesthetic				0.183
Spinal	397	325	72	
General	124	96	28	
General + regional	6	5	1	
Surgeon	527	426	101	**<0.001**
Surgical start time	9:34 ± 88 mins	9:26 ± 82 mins	10:06 ± 100 mins	**<0.001**
30-day readmission	10	7	3	0.772
6-month reoperation	11	8	3	0.477

BMI, body mass index; ASA, American Society of Anesthesiologists; TJA, total joint arthroplasty; TKA, total knee arthroplasty; THA, total hip arthroplasty.

Note: values in bold indicate statistical significance.

### Causes of failed discharge

Of the study cohort, 101 (19%) patients failed SDH discharge and required inpatient admission postoperatively (Table 3). The predominant cause of a failed discharge was orthostatic hypotension followed by ineligible patients that precluded them from being discharged same day based on established institutional guidelines.

**Table 3.** Causes of failed discharge.

Cause	Rate
Medically unstable	50 (50%)
Orthostatic hypotension/syncopal episode	20
Urinary retention	9
PONV	8
Supplemental O2 requirements	7
Hypertension	3
Other	3
Not eligible for SDH	19 (19%)
Pain	14 (13%)
Falls risk	10 (10%)
Oxygen monitoring required	8 (7%)

PONV, postoperative nausea and vomiting; SDH, same-day home.

### Bivariate analysis

There was a significant difference in preoperative ASA classification between the successful and failed SDH groups (Χ^2^ = 8.86; *p* = 0.031) (Table 2). There were significant differences in failed discharge rates among the treating surgeons (Χ^2^ = 34.1; *p* *<* 0.001). A statistically significant difference in the proportion of patients who had received a previous TJA was found between the 2 groups (Χ^2^ = 6.18; *p* = 0.013). The proportion of patients with a THA was significantly higher in the failed SDH group (Χ^2^ = 7.22; *p* = 0.007). Finally, there was significant difference in surgical start time (9:26 ± 82 minutes vs. 10:06 ± 100 minutes; *p* *<* 0.001) between the successful and failed SDH groups. Following regression analysis, a previous TJA was not an independent predictor of successful discharge.

There was no significant difference in age, gender or BMI between the 2 groups. No differences were found in 30-day readmission or reoperations within 6 months between the 2 groups. In patients undergoing THA, there was no differences in failed discharge rates among the 3 surgical approaches utilised (Appendix 2).

### Multivariate logistic regression analysis

2 of the treating surgeons were independently associated with higher failed discharge rates in patients scheduled for SDH (Table 4). ASA class IV was found to be independently associated with a higher chance of failed SDH discharge following TJA (OR 3.4; 95% CI, 1.4–8.2; *p* = 0.006). A later surgical start time was also found to be an independent predictor of failed SDH in this cohort (OR 1.3; 95% CI, 1.15–1.55; *p* = 0.001). Finally, THAs were found to be an independent predictor of failed SDH (OR 2.0; 95% CI, 1.2–3.1; *p* = 0.004).

**Table 4.** Multivariate regression analysis.

Variable	β regression coefficient	Standard error (SE)	*p*–Value	OR (95% CI)
Surgical start time	0.291	0.075	0.001*	1.3(1.1–1.6)
Previous TJA				
Yes	Reference			
No	1.150	0.614	0.069	3.1(0.92–10.6)
Operation				
TKA	Reference			
THA	0.680	0.237	**0.003**	2.1(1.3–3.4)
Surgeon				
Surgeon 1	Reference			
Surgeon 2	0.903	0.374	**0.016**	2.5(1.2–5.1)
Surgeon 3	1.8	0.514	**<0.001**	6.1 (2.2–16.6)
Surgeon 4	0.708	0.431	0.101	2.0(0.87–4.7)
Surgeon 5	0.396	0.417	0.343	1.5(0.66–3.7)
Surgeon 6	0.261	0.369	0.480	1.3(0.63–2.7)
ASA				
I	Reference			
II	0.007	0.309	0.983	1.0(0.56–1.8)
III	0.28	0.278	0.312	1.3(0.77–2.3)
IV	1.245	0.454	**0.006**	3.5(1.4–8.5)

TJA, total joint arthroplasty; TKA, total knee arthroplasty; THA, total hip arthroplasty; ASA, American Society of Anesthesiologists; OR, odds ratio; CI, confidence interval.

Values in bold indicate statistical significance.

## Discussion

Despite SDH pathways, a significant proportion of patients who are scheduled for SDH discharge still require inpatient admission postoperatively. In the present study, in a consecutive cohort of 527 patients scheduled for SDH TJA, 19% of patients failed discharged requiring admission to hospital postoperatively. The most common cause was medically unstable patients followed by patients inappropriately booked for a SDH discharge. Individual surgeons, later surgical start time, ASA IV class, and undergoing a THA were all independent predictors of failed discharge after multivariate regression analysis.

An important finding from the current study is that nearly 20% of patients scheduled for SDH were not eligible based on our institutional guidelines. Barriers for failed discharge in this population included known OSA requiring oxygen monitoring, late OR start times or no support person in place. This represents an area of improvement to reduced failed discharges that can be easily implemented.^16^ Effective interdisciplinary communication can be utilised to ensure all team members of the SDH pathway are aware of the contraindications to SDH surgery and flag patients who are ineligible.

Perhaps the most important finding of the current study is the significant variability in failed discharge rates among treating surgeons. It emphasises the importance of appropriate patient selection and preoperative patient education and suggest that there is a skill to achieving successful SDH discharge in this population. London et al.^17^ demonstrated that surgeons who actively utilised a preoperative discharge planning protocol were more likely to discharge their patients home as opposed to inpatient rehabilitation or convalescent care. Another potential surgeon-related variable is the use of perioperative adjuncts such as periarticular injections, nerve blocks, cryotherapy, preoperative patient education, counselling prior to surgery and surgeon-patient discussion regarding patient preference to be discharged same day.^8,17^ These adjuncts are useful tools in successfully managing postoperative pain and rapid recovery and variability among surgeons in this cohort may have contributed to different success rates.^18^ Unfortunately, our database did not accurately capture these discrete variables. These findings highlight the importance that not only patient education, but surgeon education, is critical to the success of a SDH TJA programme.

In this cohort, a later surgical start time was found to be an independent risk factor for a failed SDH discharge. This is intuitive as earlier surgical times allow for enough time for the patient to recover from anaesthesia, receive postoperative education from the nursing staff and pass mobility testing with the physiotherapy team. Later surgical start time has been found to be a predictor of failed discharge in other cohorts and proposed SDH TJA pathways mandate that scheduled SDH cases must be the first or second case of the day.^14,19^ However, it remains to be seen whether early surgical start time is truly a requirement of SDH surgery as many of the barriers to discharge are institutional in nature.^10^

At our centre, the physiotherapy team is available for patient assessments until 5:00 p.m. each day and patients who are not cleared by this point are admitted for physiotherapy evaluation the following morning. Cost-effectiveness studies are warranted to assess the feasibility and potential cost savings of having team members available later in the day to facilitate an evening discharge. Another potential confounding variable in this regard is the variation in anaesthetic technique in patients undergoing spinal anaesthetic.^20^ Although the use of fast-acting spinal anaesthetics was encouraged at our institution, it was not a pre-requisite for eligibility and longer acting spinals may have contributed to later surgical start times leading to failed discharges.

Consistent with the available published literature, patients classified as ASA IV were at a higher risk of failed discharge.^21^ ASA IV indicates that patients have “severe incapacitating disease process that is a constant threat to life” which suggest that these patients should have not been scheduled for a SDH procedure. This highlights the importance of communication between the anaesthesia team and the treating surgeon. Ideally, these patients are flagged at their preoperative anaesthetic visit as inappropriate candidates for SDH TJA to avoid miscommunication and a potential failed discharge on the day of surgery. Interestingly, contrary to previous literature, ASA III was not an independent predictor of failed discharge.^14,21–23^ Similarly, age, sex, BMI have all been previously associated with prolonged length of stay and failed SDH discharge but demonstrated no association in our cohort.^20,24^ Based on the results of the current cohort, ASA III, older age and BMI should not be absolute contraindications for inclusion in a SDH pathway.

Surprisingly, patients undergoing THA were twice as likely to fail discharge when compared to TKA patients. A subgroup analysis demonstrated no difference in failed discharge between surgical approaches despite differences in early recovery found in the literature.^25^ However, there were a limited number of THAs performed via the posterior approach and these results should be taken with caution. 1 potential confounding variable that was not evaluated due to the constraints of our database was selective nerve blocks. At our institution, adductor canal blocks for TKAs are utilised more regularly than pericapsular nerve group blocks for THAs which may have contributed to the difference in failed discharges.^26,27^ Given that this is a novel finding that has not been demonstrated in other cohorts, further research is required to understand causes and risks of failed discharge in this particular population.^14^

This study is strengthened by its large, consecutive cohort evaluating SDH in a single-payer healthcare system in which SDH TJA has not been widely adopted.^10^ This is a retrospective cohort study that carries inherent biases. This study represents a cohort from a high-volume tertiary arthroplasty centre which may limit its generalisability. Thirdly, some patient characteristics including number of allergies, preoperative medications and history of anxiety or depression have been associated with prolonged length of stay and failed discharge but were not in recorded in our database.^11^ Patient-reported outcomes were not reliably collected and thus could not be included in this study. Finally, both THA and TKA patients were included in this analysis despite having differing perioperative demands. However, the majority of the literature reports on both THA and TKA together and the inclusion of both cohorts allowed for a comparison to the published literature.

## Conclusion

In this review of 527 patients scheduled for SDH TKA or THA, there was a 19% failed discharge rate. The main causes for failed discharge were medically unstable patients postoperatively and inappropriate patient selection. The treating surgeon, later surgical time, ASA IV classification and patients undergoing THA were all independent predictors of failed discharge.

## Supplemental Material

sj-pdf-1-hpi-10.1177_11207000221111101 – Supplemental material for Causes and predictors of failed same-day home discharge following primary hip and knee total joint arthroplasty: a Canadian perspective Click here for additional data file. Supplemental material, sj-pdf-1-hpi-10.1177_11207000221111101 for Causes and predictors of failed same-day home discharge following primary hip and knee total joint arthroplasty: a Canadian perspective by Aaron M Gazendam, Daniel Tushinski, Mansi Patel, Kamal Bali, Danielle Petruccelli, Mitchell Winemaker, Justin DeBeer, Leslie Gillies, Katie Best, Jennifer Fife and Thomas J Wood in HIP International

sj-pdf-2-hpi-10.1177_11207000221111101 – Supplemental material for Causes and predictors of failed same-day home discharge following primary hip and knee total joint arthroplasty: a Canadian perspective Click here for additional data file. Supplemental material, sj-pdf-2-hpi-10.1177_11207000221111101 for Causes and predictors of failed same-day home discharge following primary hip and knee total joint arthroplasty: a Canadian perspective by Aaron M Gazendam, Daniel Tushinski, Mansi Patel, Kamal Bali, Danielle Petruccelli, Mitchell Winemaker, Justin DeBeer, Leslie Gillies, Katie Best, Jennifer Fife and Thomas J Wood in HIP International
