# Supplementary material for: Causes and predictors of failed same-day home discharge following primary hip and knee total joint arthroplasty: a Canadian perspective
Source: Hip Int. 2022 Jul 17;33(4):576–82. doi: 10.1177/11207000221111101 (PMC10338690; doi:10.1177/11207000221111101)
Supplement: sj-pdf-1-hpi-10.1177_11207000221111101 – Supplemental material for Causes and predictors of failed same-day home discharge following primary hip and knee total joint arthroplasty: a Canadian perspective [file sj-pdf-1-hpi-10.1177_11207000221111101.pdf]

Table 3. Causes of readmissions and reoperations.

Cause of Reoperation	Failed Discharge (n=3)	Successful Discharge (n=8)
Knee Manipulation Under Anesthesia	1	5
Surgical site infection	1	2
Periprosthetic fracture	1	1
Causes of 30-Day Readmissions	Failed Discharge (n=3)	Successful Discharge (n=7)
Reoperation	1	2
Syncopal Episode	0	2
Ileus	0	1
Uncontrolled Atrial Fibrillation	1	0
Pulmonary Embolism	1	0
Esophagitis	0	1
Addisonian Crisis	0	1
