# Supplementary material for: Causes and predictors of failed same-day home discharge following primary hip and knee total joint arthroplasty: a Canadian perspective
Source: Hip Int. 2022 Jul 17;33(4):576–82. doi: 10.1177/11207000221111101 (PMC10338690; doi:10.1177/11207000221111101)
Supplement: sj-pdf-2-hpi-10.1177_11207000221111101 – Supplemental material for Causes and predictors of failed same-day home discharge following primary hip and knee total joint arthroplasty: a Canadian perspective [file sj-pdf-2-hpi-10.1177_11207000221111101.pdf]

Table 5. THA Surgical Approach

Approach	Successful SDH	Failed	P-value
Lateral	92	32	0.312
Direct Anterior	91	23	
Posterior	11	6	
